# Modulation of Response Times During Processing of Emotional Body Language

**DOI:** 10.3389/fpsyg.2021.616995

**Published:** 2021-02-25

**Authors:** Alessandro Botta, Giovanna Lagravinese, Marco Bove, Alessio Avenanti, Laura Avanzino

**Affiliations:** ^1^Department of Experimental Medicine (DIMES), Section of Human Physiology, University of Genoa, Genoa, Italy; ^2^Department of Neuroscience, Rehabilitation, Ophthalmology, Genetics and Maternal Child Health (DINOGMI), University of Genoa, Genoa, Italy; ^3^IRCCS Policlinico San Martino, Genoa, Italy; ^4^Centro di Neuroscienze Cognitive and Dipartimento di Psicologia, Campus Cesena, Alma Mater Studiorum – University of Bologna, Cesena, Italy; ^5^Centro de Investigación en Neuropsicología y Neurociencias Cognitivas, Universidad Católica del Maule, Talca, Chile

**Keywords:** emotion, body language, reaction time, posture, IAPS

## Abstract

The investigation of how humans perceive and respond to emotional signals conveyed by the human body has been for a long time secondary compared with the investigation of facial expressions and emotional scenes recognition. The aims of this behavioral study were to assess the ability to process emotional body postures and to test whether motor response is mainly driven by the emotional content of the picture or if it is influenced by motor resonance. Emotional body postures and scenes (IAPS) divided into three clusters (fear, happiness, and neutral) were shown to 25 healthy subjects (13 males, mean age ± SD: 22.3 ± 1.8 years) in a three-alternative forced choice task. Subjects were asked to recognize the emotional content of the pictures by pressing one of three keys as fast as possible in order to estimate response times (RTs). The rating of valence and arousal was also performed. We found shorter RTs for fearful body postures as compared with happy and neutral postures. In contrast, no differences across emotional categories were found for the IAPS stimuli. Analysis on valence and arousal and the subsequent item analysis showed an excellent reliability of the two sets of images used in the experiment. Our results show that fearful body postures are rapidly recognized and processed, probably thanks to the automatic activation of a series of central nervous system structures orchestrating the defensive threat reactions, strengthening and supporting previous neurophysiological and behavioral findings in body language processing.

## Introduction

The investigation of how humans perceive and respond to emotional signals conveyed by body expressions has been for a long time secondary compared with research addressing the recognition of emotional faces or emotional scenes (De Gelder, 2009; de Gelder et al., 2010). Only in the last decades, an increased interest in whole-body expressions and their emotional correlates has started to emerge (van Heijnsbergen et al., 2007; de Gelder et al., 2010, 2015; Borgomaneri et al., 2012).

As for facial expressions, processing of emotional body postures activates brain regions involved in perceptual and affective processes such as the superior temporal sulcus, fusiform and postcentral gyrus, the amygdala, and medial prefrontal cortex (Downing and Kanwisher, 2001; Peelen and Downing, 2005; De Gelder, 2009; Peelen et al., 2010; Ross et al., 2020), as well as the mirror neuron system involved in action understanding and imitation (De Gelder et al., 2004; Bachmann et al., 2018). Furthermore, processing facial and bodily emotional expressions spontaneously induces motor mimicry in the observer (Huis In ‘t Veld et al., 2014; Ross and Atkinson, 2020), a mechanism that can contribute to accurate emotion recognition (Oberman et al., 2007; Wood et al., 2016; Borgomaneri et al., 2020a). These studies suggest that perceiving others’ emotional expressions involves a simulation of motor plans and associated sensory representations engaged when making the same expressions (Adolphs et al., 2000; Niedenthal et al., 2010; Huis In ‘t Veld et al., 2014; Paracampo et al., 2017; Ross and Atkinson, 2020), reflecting a simulation of whole-body state associated with the emotion (Ross and Atkinson, 2020).

Additionally, emotional bodily expressions strongly activate subcortical motor areas such as the caudate nucleus and putamen and several regions of the cortical motor system, with stronger (De Gelder et al., 2004; de Gelder et al., 2010; Borgomaneri et al., 2017) and faster (Borgomaneri et al., 2015b, 2017) response to threatening expressions. Such motor activations may reflect sensorimotor simulation and/or the activation of motivational tendencies which facilitate emotionally congruent behavior, with positive stimuli activating the approach tendencies and negative stimuli activating the avoidance tendencies (Lang et al., 1990; Ekman and Davidson, 1995; Lang and Bradley, 2010).

Starting from all these considerations, one could speculate that readiness of the motor system may be modulated by the presence of emotional content in body posture and by the valence of the emotion. However, behavioral data in the literature are controversial, showing increased response times (RTs) in recognizing fearful body expressions (Van den Stock et al., 2007) or anger as the most difficult emotion to categorize (Atkinson et al., 2004). Noteworthy, there are methodological issues that could explain these results, as differences in the set of images used and in the behavioral task or the level of uncertainty in categorizing the emotional stimuli.

Readiness of the motor system can be studied by means of neurophysiological techniques in addition to behavioral paradigms. Recently, Borgomaneri and coworkers developed a novel set of visual stimuli in order to test the activity in the motor cortex during processing of emotional body postures and trying to address the aforementioned methodological issues (Borgomaneri et al., 2012). Results showed that only fearful body expressions were able to modulate cortical excitability at a very early stage of emotional processing (between 70 and 150 ms after stimulus onset) (Borgomaneri et al., 2015a,b,c, 2017, 2020b). However, whether this corresponds to a modulation of motor behavior has not been addressed so far.

Therefore, the first aim of the present study was to investigate if there was a specific modulation of motor response during processing of emotional body postures by assessing RTs in a three-alternative forced choice task using the set of visual stimuli adopted by Borgomaneri and colleagues. Based on previous works and taking into account the considerations we previously made about the similarities between the processing of emotional body language and facial expressions and their respective RTs which resulted faster for happiness, we expected that the motor response to fearful body expressions would have been longer relative to happy and neutral body expressions, with shorter RTs for positive stimuli (Buodo et al., 2002; Van den Stock et al., 2007; Calvo and Beltrán, 2013; Borgomaneri et al., 2015c).

Our second objective aimed to compare RTs during processing of emotional body postures with RTs during processing of emotional scenes, in order to test whether reactivity to fear-related signals is specific to the observation of human bodies. Specifically, in the second aim, RTs in a three-alternative forced choice task were recorded using emotional pictures taken from the International Affective Picture System (IAPS). The use of IAPS pictures compared with other sets of stimuli gives the opportunity to manipulate the arrays of images used in the experiment, matching the values of valence and arousal of the two groups of pictures that are being used and controlling the new set of images (e.g., emotional postures) in order to accurately investigate whether the effects of the exposure may be driven by the stimulus features or by their intrinsic emotional properties (Calvo and Avero, 2009).

Yet, studies comparing emotional scenes and facial expressions have shown that brain regions involved in emotional processing are more sustainably activated and RTs in a categorization task are faster when processing faces relative to the IAPS (Atkinson et al., 2004). These findings have been related to the greater complexity and novelty of emotional scenes—which may require larger cognitive load and consequently slower RTs relative to faces. On the other hand, some consistent features displayed by facial expressions (e.g., eyes, nose, mouth) could lead to a faster habituation (Britton et al., 2006).

Based on these premises and considering the similarities between the underlying features of face and body images, we expected to find longer RTs for IAPS pictures, mainly explicable by their intrinsic complexity. Moreover, considering the high amount of motor information inherently depicted in body language, we also had to consider motor contagion as a trigger to a faster response potentially increasing the temporal gap in RTs between postures and emotional scenes, unless motor response shows to be mainly driven by the emotional content rather than by the motor information (Borgomaneri et al., 2012).

## Materials and Methods

### Participants

In order to estimate an appropriate sample size, a power analysis was run based on the data retrievable in the work by Van den Stock et al. (2007). Analysis run on G^∗^Power 3.1 for comparisons of means from dependent groups with 1 − β = 0.80, α = 0.05, and an effect size of 0.62 resulted in an ideal sample size of 23 subjects. Twenty-five healthy subjects (13 males, 12 females, mean age ± SD: 22.3 ± 1.8 years) were enrolled in the study. All participants were self-reportedly right-handed and participated to both trials (postures and IAPS) in the same experimental session.

### Visual Stimuli

A total of 90 emotional visual stimuli were used in the experimental session: 45 for the emotional body language condition and 45 taken from the IAPS database as control (Lang et al., 2008). The emotional posture pictures were selected from a validated database (Borgomaneri et al., 2012, 2015c). Body language pictures depict four actors in different postures with emotional and non-emotional valence, 30 portraying negative (fear) and positive (happiness) movements and 15 with no emotional significance (neutral). The actors were not handling objects and their faces were blanked out. The luminance and refresh rate of pictures were controlled and matched for all images *via* a photosensor (data processed *via* E-Prime 3.0).

Regarding the IAPS pictures, 45 stimuli were taken from the IAPS database (Lang et al., 2008): 15 with negative emotional valence (fear), 15 with positive valence (happiness), and 15 neutral pictures (neutral). All the pictures were mirrored alongside the vertical axis in order to obtain 90 stimuli per trial, implementing the data pool while avoiding the repetition of the same stimulus.

Some issues emerged during the selection of the stimuli. Firstly, fearful emotional postures were emotion-specific; in other words, the body expression depicted in the pictures was unequivocally related to the pure emotion “fear” (Ekman, 1999; Borgomaneri et al., 2012; Huis In ‘t Veld et al., 2014). On the other hand, “negative” pictures in the IAPS database often show several aversive emotions combined (e.g., fear and disgust or fear and sadness). In order to avoid this possible bias, we selected the IAPS stimuli for the fear condition from a restricted sample of pictures that have been reported to mainly evoke fear (e.g., human attacks and accident-depicting pictures) (Barke et al., 2012). Secondly, in order to exclude most of the body movement information, we decided to exclude all IAPS pictures that depicted whole human bodies involved in some kind of actions.

With regard to the other two conditions, happiness and neutral, we did not find particular differences or risk of bias in the recognition of the intrinsic emotional valence of pictures. We decided to include only pictures of families and babies and “adventures” in the happiness condition with partial or no human body representation. In order to strengthen the aforementioned assumptions, after the experiment, we also submitted a questionnaire to each subject for both postures and IAPS, in order to evaluate emotional content, valence, and arousal of each stimulus.

### Task

**FIGURE 1:**
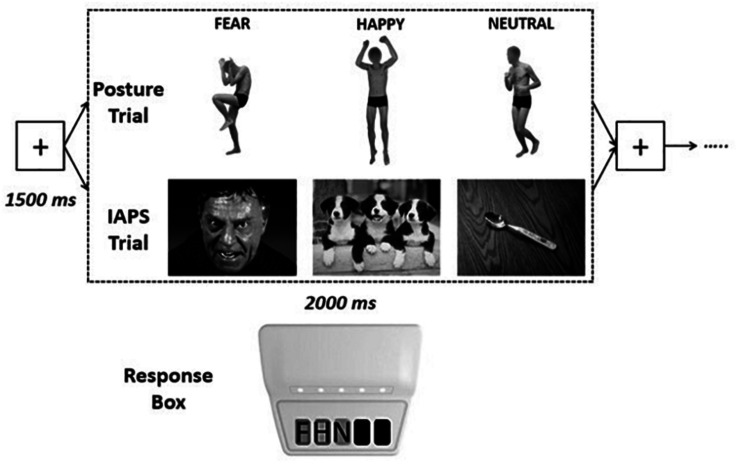
Experimental design. Each visual emotional stimulus had a maximum duration of 2,000 ms [the stimulus disappeared as soon as the participants pressed one of the keys of the response box, recording the response and the response time (RT)], interspersed by a fixation cross screen of 1,500 ms, for a total of 90 stimuli for each trial [postures or International Affective Picture System (IAPS)]. Response times were recorded by pressing one of three keys on the Chronos response box with: key 1 = negative (F), key 2 = positive (H), key 3 = nonemotional (N).

Visual stimuli were presented on a 22-in. computer screen (resolution: 1,680 × 1,050, refresh rate: 60.0 Hz; 16.67 ± 12.37 ms) located at 80 cm away from the subjects. Refresh rate was assessed *via* a photosensor connected to the response box and corresponded to normative values (Garaizar et al., 2014). Stimuli were presented using the E-Prime 3.0 software (Psychology Software Tools, Pittsburgh, PA, United States). The order of presentation was randomized, and each stimulus had a maximal duration of 2,000 ms, with an interstimulus interval fixation screen of 1,500 ms (see Figure 1). Participants were asked to keep the right hand on a USB-based data collection device named Chronos (Psychology Software Tools, Pittsburgh, PA, United States), with the second, third, and fourth finger on the first, second, and third key of the response box, respectively. They were asked to categorize each visual stimulus as negative, positive, or neutral by pressing one of the three buttons, with different stimulus–response combinations across participants. RTs were taken as the difference in milliseconds between the onset of the visual stimulus and the pressing of the key on the response box.

### Stimuli Evaluation

Participants were presented with all the 90 stimuli (45 body postures and 45 IAPS stimuli) and asked to evaluate them using a questionnaire with no time pressure. Participants were first asked to recognize the emotion depicted in the picture by choosing between seven options: fear, sadness, disgust, anger, surprise, happiness, and neutral. We considered as correct only three out of the seven options, which are fear, happiness, and neutral. The other choices were not taken into consideration because of being outside of the study’s main interest. We then asked the participants to rate valence on a Likert scale ranging from 1 to 9, where 1 indicated “absolutely unpleasant” and 9 indicated “absolutely pleasant”; they used a similar scale to evaluate arousal, where 1 indicated “no arousal” and 9 indicated “high arousal.”

### Experimental Design and Procedure

Subjects were comfortably sitting on a chair at a distance of approximately 80 cm from the computer screen where the visual emotional stimuli were presented. Participants were asked to keep the right hand on the Chronos device. After a brief explanation of the task and the presentation of a first, fixed screen with the instructions to follow during the trial, participants were asked to press a key in order to start with a five stimuli test trial in order to familiarize with the task before starting the complete 90 stimuli experimental trial.

The experiment was divided into two sessions, one with the emotional postures and one with the IAPS pictures, in which the order was randomized in order to exclude the familiarization with the task in one particular trial and ended with questionnaires’ filling.

### Data Analysis

For each body posture and IAPS category, we computed an accuracy index as the percentage of correct responses in the forced three-choice task. Pictures with an accuracy lower than 80% were excluded from further analyses (three happiness postures and three neutral postures for body stimuli and three neutral pictures for IAPS stimuli).

#### Accuracy

A 2 × 3 repeated-measures analysis of variance (rmANOVA) with PICTURE (posture and IAPS) and CATEGORY (negative, positive, and neutral) as main effects was performed on the accuracy in the recognition of the stimuli during the forced choice RT task.

In the categorization task included in the questionnaires, a 2 × 3 rmANOVA with PICTURE (posture and IAPS) and EMOTION (fear, happiness, and neutral) as within-subject factors was performed on accuracy data for both postures and IAPS conditions.

In order to be analyzed *via* an rmANOVA, accuracy data were transformed to arcsine values.

#### Valence and Arousal

On valence and arousal data, a logarithmic transformation was performed in order to normalize the data distributions. *Post hoc* analysis was performed on significant effects *via* Bonferroni correction of significance. Valence and arousal data were analyzed performing a 2 × 3 rmANOVA with PICTURE and EMOTION as within-subject factors.

#### Response Times and Their Coefficient of Variation

RTs were analyzed performing a 2 × 3 rmANOVA with PICTURE and CATEGORY as within-subject factors. Only trials in which categorization in the forced choice RT task was correct were considered for the analysis of RT data and only if they fell in between two standard deviations from their respective mean. The coefficient of variation (CV) of RTs was computed as the standard deviation of RTs divided by the mean of RTs for each emotion in both postures and IAPS.

#### Correlations and Reliability Analysis

Correlations between valence and arousal were analyzed by means of Pearson’s correlation coefficient for normally distributed data, whereas nonparametric Kendall’s tau correlation method was used in conditions of non-normality. Bonferroni correction was then performed for multiple comparisons. Correction on significance was calculated taking into account multiple comparisons for valence and arousal, meaning that the correction on α was 0.025.

Cronbach’s alpha coefficient was then studied to assess the reliability of the visual stimuli used in the experiment.

Statistical analysis was performed *via* SPSS Statistics 23.0 (IBM, Somers, United States). The significant level was set at α = 0.05. Normality was tested *via* the Shapiro–Wilk test and violations of sphericity were corrected through the Greenhouse–Geisser method.

## Results

### Accuracy

Accuracy in the three-alternative forced choice RT task was high (∼94%). The rmANOVA showed a significant main effect of PICTURE (*F*_1,24_ = 7.907; *p* = 0.01; *pη*^2^ = 0.248), with a higher accuracy observable for IAPS compared with posture (94.8 ± 1.4 and 92.6 ± 1.6%, respectively), but no main effect of CATEGORY or PICTURE^∗^CATEGORY interaction (*F* < 1 and *p* > 0.05). Descriptive statistics are reported in Table 1.

**TABLE 1:** Descriptive statistics.

	Postures			IAPS		
		
	Negative	Positive	Neutral	Negative	Positive	Neutral
Accuracy (RT task) (mean ± SD)	93.47 ± 5.57	92.53 ± 8.18	91.73 ± 10.05	95.60 ± 5.16	93.20 ± 8.02	95.73 ± 8.25
Reaction times (mean ± SD)	759.04 ± 144.38	868.71 ± 114.24	897.34 ± 168.49	716.31 ± 102.48	696.94 ± 112.96	712.64 ± 100.11

*In the table, reported are all response times (RTs) recorded during the three-alternative forced choice task and the respective accuracy for all the subjects. All values are reported as mean ± SD. IAPS, International Affective Picture System.*

Questionnaire data showed lower accuracy, particularly with fear IAPS pictures (∼82% of correct answers). The rmANOVA showed a significance a main effect of EMOTION (*F*_2,48_ = 14.282; *p* < 0.01; *pη*^2^ = 0.373) indicating that accuracy was lower with fear (85.9 ± 3.0%) compared with happiness (91.3 ± 2.5%; *p* = 0.05) and neutral stimuli (96.2 ± 1.6%; *p <* 0.01) and lower with happiness compared with neutral stimuli (*p* = 0.05). Moreover, the PICTURE^∗^EMOTION interaction was significant (*F*_2,48_ = 4.870; *p* = 0.01; *pη*^2^ = 0.169) accounted for by reduced accuracy in the fear IAPS stimuli condition. *Post hoc* analysis showed that accuracy for fear IAPS stimuli (81.6 ± 3.0%) was significantly lower compared with happiness IAPS (93.3 ± 1.5%; *p* < 0.01) and neutral IAPS stimuli (97.1 ± 1.6%; *p* < 0.01) and lower than fear posture stimuli (90.1 ± 3.0%; *p* = 0.02); no other significant differences were observed (all *p* > 0.05). For details, see Table 2.

**TABLE 2:** Descriptive statistics.

	Postures			IAPS		
		
	Fear	Happiness	Neutral	Fear	Happiness	Neutral
Accuracy (questionnaires) (mean ± SD)	90.13 ± 14.92	89.33 ± 17.43	95.33 ± 10.51	81.60 ± 15.19	93.33 ± 7.45	97.07 ± 5.80
Valence (mean ± SD)	2.41 ± 0.94	7.38 ± 0.86	5.00 ± 0.14	2.09 ± 0.67	7.32 ± 0.76	5.11 ± 0.29
Arousal (mean ± SD)	6.08 ± 1.94	6.26 ± 1.68	1.61 ± 1.15	7.20 ± 0.97	5.88 ± 1.33	1.575 ± 1.17

*In the table, reported are all valence and arousal values as well as the accuracy recorded during the questionnaires submitted to the participants. All values are reported as mean ± SD.*

### Valence and Arousal

Mean values for valence and arousal are reported in Table 2. The rmANOVA on valence data showed a significant main effect for EMOTION (*F*_2,48_ = 298.278; *p* < 0.01; *pη*^2^ = 0.926), while no significance was found for the effect of PICTURE and PICTURE^∗^EMOTION (all *F* < 1 and *p* > 0.05). As expected, *post hoc* analysis showed lower valence values for fear stimuli compared with happiness and neutral stimuli (all *p* < 0.01) and higher valence values for happiness compared with neutral stimuli (*p* < 0.01).

The rmANOVA on arousal data showed a significant main effect of EMOTION (*F*_2,48_ = 258.971; *p* < 0.01; *pη*^2^ = 0.915), but not of PICTURE (*F* < 1 and *p* > 0.05), and a significant PICTURE^∗^EMOTION interaction (*F*_2,48_ = 3.449; *p* = 0.04; *pη*^2^ = 0.126). *Post hoc* analysis on the main effect of EMOTION showed higher values for fear stimuli compared with happiness (*p* = 0.02) and neutral (*p* < 0.01) and higher values for happiness stimuli compared with neutral stimuli (*p* < 0.01) (see Table 2). As for the interaction effect, greater arousal was found for fear IAPS stimuli than for happiness IAPS and neutral IAPS stimuli (*p* < 0.01) and for happiness IAPS relative to neutral IAPS stimuli (*p* < 0.01). Fear IAPS stimuli also showed greater arousal values than fear posture stimuli (*p* = 0.01), whereas no difference between picture types was found for the other two emotion categories (all *p* > 0.05). Fear posture and happiness posture had greater arousal values than neutral posture (*p* < 0.01) but did not differ from one another (*p* > 0.05).

### Response Times and Their Coefficient of Variation

**FIGURE 2:**
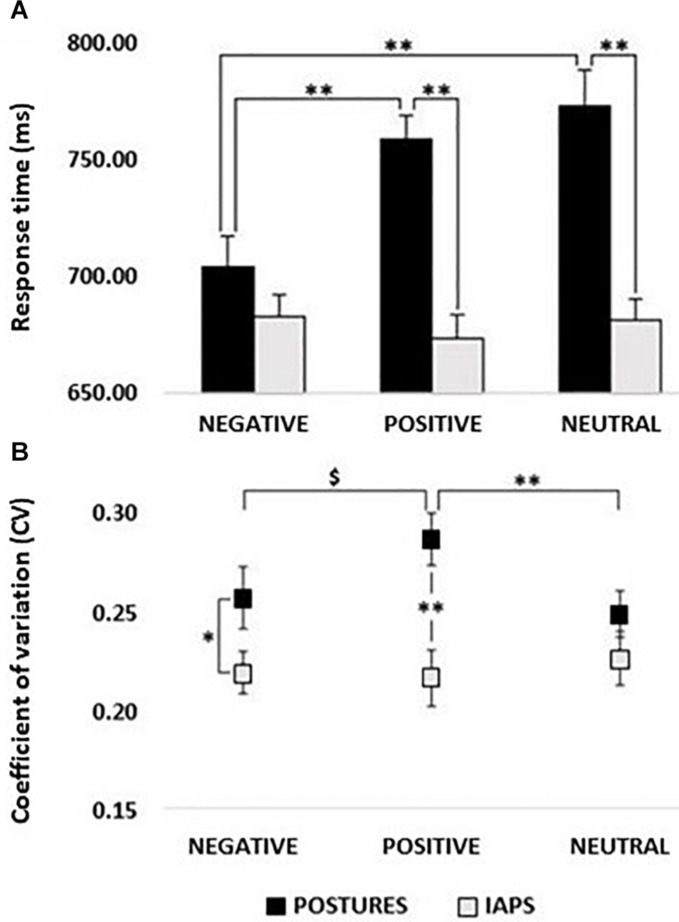
**(A)** Response times (RTs). Histogram showing a comparison between RTs recorded in postures and IAPS trials. Significant differences were found between negative and positive and negative and neutral for postures, as well as between positive and neutral in the comparison between postures and IAPS, but not for negative. No differences were retrievable between emotions in the IAPS trial. RT values in milliseconds (ms) are reported in the *y*-axis, and emotions are reported on the *x*-axis with black bars for postures and gray bars for IAPS. **(B)** Coefficient of variation; postures vs IAPS. Positive’s CV in the posture trial was significantly higher than the one computed for neutral and showed a trend in the comparison with negative, while no differences were found for IAPS. Postures’ negative and positive CVs were higher in the comparison with the variation retrieved in the IAPS trials. CV ranges from 0 to 1; on the *y*-axis, it is possible to observe a partial scale that focuses on the range of the CV found in the experiment. Emotions are reported on the *x*-axis with a straight line for postures and a dotted line for IAPS. Legend: ^∗^ = *p* < 0.05, ^∗∗^ = *p* < 0.01, $ = *p* > 0.05.

The rmANOVA on RTs showed the main effect of PICTURE (*F*_1,24_ = 27.333; *p* < 0.01; *pη*^2^ = 0.532) with lower RTs for IAPS compared with postures and the main effect of CATEGORY (*F*_2,48_ = 4.881; *p* = 0.02; *pη*^2^ = 0.169) with lower RTs for negative stimuli compared with positive and neutral stimuli (all *p* < 0.01), but no differences between positive and neutral stimuli (*p* > 0.05). Remarkably, the PICTURE^∗^CATEGORY interaction was also significant (*F*_2,48_ = 12.076; *p* < 0.01; *pη*^2^ = 0.335). *Post hoc* analysis showed lower RTs for negative posture relative to positive posture and neutral posture (all *p* < 0.01) which in turn did not differ from one another (*p* > 0.05). Moreover, no significant differences were found between IAPS emotion categories (*p* > 0.05; see Figure 2A). In the comparison between picture types (posture vs. IAPS), positive and neutral posture stimuli had slower RTs than positive and neutral IAPS stimuli (all *p* < 0.01), but no differences were found between negative posture and negative IAPS stimuli (*p* > 0.05; for details, see Table 1 and Figure 2A).

The CV of RTs ranged between 20 and 30% (Figure 2B). The rmANOVA on CV showed a significant main effect of PICTURE (*F*_1,24_ = 9.292; *p* < 0.01; *pη*^2^ = 0.279) with higher CV values for posture than for IAPS stimuli. Moreover, a significant PICTURE^∗^CATEGORY interaction (*F*_2,48_ = 4.436; *p* = 0.03; *pη*^2^ = 0.156) showed higher CVs for posture compared with IAPS in the negative (*p* = 0.03) and positive condition (*p* < 0.01), but not in the neutral condition (*p* > 0.05). Moreover, for the posture category, positive stimuli had significantly larger CV compared with neutral (*p* = 0.01) and marginally larger CV compared with negative stimuli (*p* = 0.07) which in turn did not differ from one another (*p* > 0.05).

### Correlations

**FIGURE 3:**
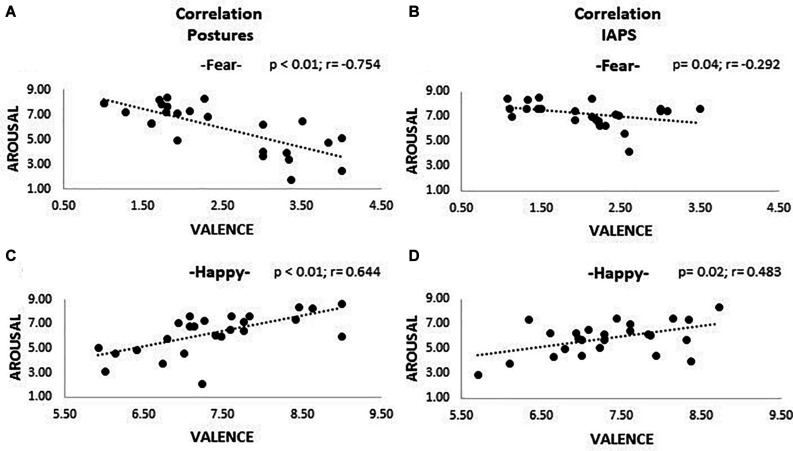
Correlation plots. The picture shows four plots where correlations between valence and arousal are observable. Plots **(A)** and **(C)** show the results for the posture trials; **(B)** and **(D)** the ones for the IAPS trials. A positive correlation is appreciable for positive emotional pictures (happiness; plots **C** and **D**), meaning that higher valence corresponds to higher activation; the opposite is observable for negative pictures (fear; plots **A** and **B**), even though for IAPS, stimuli significance was not reached after Bonferroni correction. Both valence and arousal are reported as values on the 0–9 Likert scale. Legend: $ = *p* > 0.05.

Correlations between valence and arousal ratings are shown in Figure 3. After applying the Bonferroni correction, all correlations found for happiness pictures survived, showing larger arousal for high-valence stimuli both for posture (*r* = 0.644, *p* < 0.01) and IAPS categories (*r* = 0.483, *p* = 0.02). Regarding fear stimuli, we found a significant negative correlation for posture (*r* = −0.754, *p* < 0.01), and after correction for multiple comparisons, only a marginal trend was retrievable for the IAPS stimuli (*r* = −0.292, *p* = 0.04). No correlations were retrievable in the analysis of non-emotional stimuli.

### Reliability Analysis and PCA

Item analysis on visual stimuli showed an overall good item reliability on valence and arousal ratings for all conditions except the IAPS neutral stimuli (Table 3). Good reliability is considered when Cronbach’s alpha is greater than 0.8, excellent reliability when α > 0.9, acceptable when α > 0.7, questionable when α > 0.6, poor when α > 0.5, and unacceptable when α < 0.5 (Gliem and Gliem, 2003). In order to verify the feasibility of the item analysis and the adequacy of the sample size, eigenvalues of each component were calculated *via* a principal component analysis (PCA) for both valence and arousal ratings in all conditions (fear, happiness, and neutral). For small samples, a first eigenvalue (*λ*_1_) of at least 6 is considered optimal in order to calculate a valid Cronbach’s alpha coefficient, and eigenvalues between 3 and 6 have to be considered acceptable but it means that the sample size should be increased in order to give a completely unbiased coefficient (Halil, 2008). Eigenvalues for valence and arousal ratings of body postures showed adequate first eigenvalues (all *λ*_1_ ≥ 6) for all conditions, meaning that the sample size was correctly estimated in order to verify the reliability of the visual stimuli for emotion and nonemotion detection. The IAPS stimulus showed weaker results with 4 ≤ *λ*_1_ ≤ 6, with the only exception for arousal rating of neutral stimuli where *λ*_1_ was higher than 6.

**TABLE 3:** Cronbach’s alpha for valence and arousal.

	Postures			IAPS		
		
Cronbach’s alpha	Fear	Happiness	Neutral	Fear	Happiness	Neutral
Valence	α = 0.93	α = 0.88	α = 0.75	α = 0.84	α = 0.77	α = 0.45
Arousal	α = 0.93	α = 0.93	α = 0.92	α = 0.78	α = 0.86	α = 0.97

*The table shows the Cronbach’s alpha computed in order to estimate the reliability of the two sets of images used in the experiment. An overall good reliability is observable for posture’s valence as well as for valence for fear and happiness in the IAPS trial. The only exception is observable for IAPS neutral pictures, where reliability for valence is low. Overall reliability for arousal ranges from excellent to good in all emotions and conditions.*

## Discussion

The first aim of this study was to assess the capacity to process emotional body postures in a three-choice categorization task. Our findings show significantly lower RTs for pictures depicting fearful (i.e., negative) body postures when compared with happy (i.e., positive) or neutral postures, suggesting a faster processing of fearful body language.

These results appear in contrast with prior studies investigating the processing of emotional facial expressions, where shorter RTs for positive expressions were shown and also with respect to previous behavioral studies on emotional body language (Van den Stock et al., 2007; Calvo and Beltrán, 2013; Nummenmaa and Calvo, 2015). Regarding facial processing, this difference in RTs might be explained by peculiar features that are retrievable only in happy faces. As proposed by Ekman and Friesen in 1982, happy faces are characterized by an increased bilateral activation of the zygomatic major muscles, resulting in what is commonly known as “smile,” which makes the facial expression easily recognizable and hardly misunderstood (Ekman and Friesen, 1982; Frank et al., 1993; Ekman, 1999; Calvo and Nummenmaa, 2008; Calvo and Beltrán, 2013). These features have a major role in driving the so-called “positivity offset” that leads to faster and more accurate processing of positive facial expressions (Calvo and Beltrán, 2013).

In relation to emotional processing of body postures, evidence regarding body language is not so straightforward and shows contrasting results: if on one side neurophysiological studies investigating the processing of emotional body postures have shown faster modulation of motor excitability when observing negative emotions, behavioral studies on RTs showed that motor responses are slower for negative whole-body expressions and faster for the positive ones (De Gelder et al., 2004; Van den Stock et al., 2007; Borgomaneri et al., 2012, 2015c; Huis In ‘t Veld et al., 2014).

Thus, it appears that there is incongruency between neurophysiological responses and motor outcomes, with the former apparently driven by a “negativity bias” and the latter by an advantage of positive postures, similar to emotional facial expressions (Cacioppo and Berntson, 1999; De Gelder et al., 2004). It may be argued that this mismatch might be a consequence of the fact that body postures seem not to have peculiar and unambiguous physical features such as the smile that might propend for a positive evaluation so that it is harder to extract precise information on the emotional valence of body language, augmenting the probability to misinterpret it. Furthermore, the variety of basic negative emotions might be considered as another potential confounder in the detection of a specific emotion, as documented in a study by Van den Stock and colleagues where the authors found reduced accuracy in recognizing negative body postures with respect to positive ones (Borgomaneri et al., 2020a).

However, studies have also shown that the amount of information carried by postures is as fundamental and complete as the ones deducible from facial expressions (Meeren et al., 2005; Aviezer et al., 2012; Ross and Flack, 2020), and recent models of emotion recognition suggest that the perception of negative expressions in others is able to trigger internal emotional states, which consequently yield to motor responses (i.e., activation of facial or postural muscles) and favor emotional recognition [for a review, see Ross and Atkinson, 2020]. These considerations, together with the neurophysiological modulation derived from negative emotional processing highlighted above and some methodological considerations that will follow, lead then to a possible explanation of our results.

First of all, in contrast to prior behavioral studies investigating motor response to emotional postures (Van den Stock et al., 2007; Huis In’t Veld et al., 2014), in our study, we used a set of body stimuli associated with high recognition accuracy (>90%) and no differences between posture types. This was confirmed both in the forced choice RT task where participants were asked to categorize each visual stimulus as showing negative, positive, or emotionally neutral content by pressing one of the three buttons and in the subsequent questionnaire at the end of the experimental session, where they had to categorize the posture using a wider set of emotional categories (including anger, disgust, fear, sadness, surprise, and happiness). This suggests that, in general, our images were adequately selected in order to illustrate negative or positive emotions as well as neutral stimuli. Furthermore, the analysis on accuracy data for the categorization task included in the questionnaires (see Table 2) clearly showed that the body language stimuli we selected were not only recognized as negative, positive, or neutral, but they were also correctly identified as belonging to basic emotional states (i.e., fear, happiness, or a neutral state), which prompt us to speculate that our findings could be better ascribed to specific emotional attributes rather than being driven by more general valence effects—although further studies including more emotional postures should be used to address this hypothesis.

In relation to the link between fearful stimuli and motor readiness, our results are consistent with others present in the literature. Fearful body language processing was shown to be linked to action preparation, simulation, and execution leading to an early pre-activation of postural and upper and lower limb muscles involved in the emotion observed or to facial muscles involved in the emotion (Huis In ‘t Veld et al., 2014; Ross and Atkinson, 2020). Viewing of fearful postures is shown to have an effect on the motor system at an early time (∼70–120 ms), where a suppression of intracortical facilitation of the primary motor cortex and reduced corticospinal excitability are observable, suggesting that the motor cortex may undergo a “freezing-like” phenomenon (Borgomaneri et al., 2015a,b,c, 2017, 2020b). Recent evidence on defensive threat reactions show that freezing is not a passive state but rather a parasympathetic brake on the motor system, relevant to perception and action preparation (Gladwin et al., 2016; Roelofs, 2017; Hashemi et al., 2019). Freezing has been conceptualized as an active action preparatory state with a parasympathetic driven “brake” involving the amygdala and the brainstem (periaqueductal gray) followed by a rapid adaptive response once the brake is “released” by the frontal–amygdala connections (Griebel et al., 1996; Walker and Carrive, 2003; Mobbs et al., 2007).

The open question might then be whether the motor response we found was a consequence of the emotional content of the stimuli we used or a synergic effect of emotion and motor resonance due to the intrinsic movement information expressed inherently in whole-body pictures. To address this issue, the second aim of our experiment was to assess differences in motor response between emotional body postures and IAPS.

First of all, valence and arousal ratings were comparable across the two sets of images with an exception for fear stimuli, which showed higher arousal for IAPS relative to the posture stimuli (see Table 2). Also, in both sets, Pearson’s coefficients showed a similar trend for emotion-matched stimuli, with a negative correlation between valence and arousal for fear and a positive correlation for happiness. In sum, accuracy and ratings data show that the pictures selected for both experiments were sufficiently well matched, ruling out that increased perceptual discrimination or attention allocation related to high-arousing stimuli could explain the speed up effects we observed on the RTs (Hajcak et al., 2006; Pourtois et al., 2013). The differences found for fearful stimuli do not contradict this statement because the high arousal rating of both negative postures (6.08 ± 1.94) and IAPS (7.20 ± 0.97) makes all negative pictures belonging to the category of high-arousing stimuli (Lang et al., 2008). Moreover, the item analysis that was run on the new set of emotional body postures stimuli showed an overall good reliability for valence and an excellent reliability for the arousal ratings. Thus, each stimulus was correctly recognized as fearful, happy, or neutral by all participants with an overall arousal rating higher than 5 (high-arousing stimuli) for emotional and low arousal for non-emotional pictures. This result, together with the lower error rate in emotional content categorization for emotional body language, suggests that all the stimuli depicting body language used in this experiment were equally valid and reliable in inducing a specific emotion in all participants as IAPS stimuli, if not even more adequate in evoking a response to basic emotions. Under these premises, we can consider the risk of bias linked to basic differences in categorization and misinterpretation of the emotional content of the stimuli to be low, which leads to our secondary findings.

Our data showed that RTs were longer for positive and neutral body posture in respect to positive and neutral IAPS, whereas no difference was found between negative stimuli. An explanation for this result might be linked to the capacity of negative stimuli to allocate attentional resources more rapidly compared with positive and neutral stimuli (van Heijnsbergen et al., 2007; Olofsson et al., 2008). Negative IAPS and body posture images have been shown to modulate early components of event-related potentials already after 100 ms from stimulus onset, showing a rapid allocation of attentional resources with a time course similar to that observed for emotional facial expressions (van Heijnsbergen et al., 2007; Olofsson et al., 2008). Such “negativity bias” for negative scenes and postures has been reported for brain regions involved in emotion processing (e.g., the amygdala, the orbitofrontal cortex, or the insula) and motor areas involved in motor representation and planning (e.g., premotor cortex, supplementary motor area, and parietal cortex) and the primary motor cortex (De Gelder et al., 2004; Borgomaneri et al., 2012, 2014, 2015c). The early perceptual categorization in favor of negative emotional body language associated with the early activation of motor and nonmotor neural circuits might be the reason why there are no differences in RTs between postures and IAPS and also validates the hypothesis that RTs are primarily driven by the emotional content of the observed picture rather than by the motor information carried by body postures.

On the other hand, there might be also another explanation that potentially raises the issues of comparability of the two sets of pictures and their respective complexity. IAPS pictures present more visual information compared with postures (e.g., more colors and different subjects, objects, and contextual information), and in principle, they may contain more elements to disambiguate the emotional content including facial expressions, resulting in shorter RTs. However, this possible explanation alone cannot account for the entire pattern of RT data we observed. Indeed, RTs to negative body postures did not differ from negative IAPS, suggesting similar processing speed and resource allocation for the two negative categories. On the other hand, CV data speak in favor of a higher complexity of body postures, with larger variability in RTs for body postures than for IAPS stimuli. A possible argument in opposition to this statement might occur if we look at CVs for neutral pictures; while there is no difference in RTs’ variability, motor response to body postures is significantly longer compared with IAPS. Low CV paired with longer RT may be an indication for low uncertainty in processing the image, but it also means that the image needed more time to be correctly interpreted and categorized. This might be explained by the fact that our neutral body postures showed an actor performing non-emotional actions (i.e., higher complexity) which might have led to longer times in order to correctly categorize the stimulus, but also to a low uncertainty whenever the stimulus was correctly processed. Conversely, the fact that negative postures showed higher variability compared with IAPS, no differences in RTs may be a sign that, although there might be greater uncertainty in deciphering negative body language, the motor response is accelerated, resulting in the prioritization of attentional allocation when observing a potential threat. It is also true that our results show higher CV and RTs for happy body posture compared with the ones recorded for emotional scenes, and this might suggest that they resulted to be too complex or ambiguous to be rapidly processed, but the fact that the accuracy in detecting the correct emotion in the categorization task is extremely high serves as a counterproof. A possible explanation for this finding might be that the information conveyed by the arms and hands in emotional body language are crucial in order to correctly process some specific emotions (Dael et al., 2012; Ross and Flack, 2020). A closed fist might be an indication of anger (Dael et al., 2012; Calbi et al., 2020), and consequently, it might need more time to correctly interpret the whole-body posture observed. Considering the fact that the hands draw attentional resources in interpreting the mood expressed in body language and that our happy stimuli depicted mainly the actor with closed fists but in pleasant postures (e.g., jubilation or exultation), it might be plausible to infer that the higher variability and, consequently, the longer RTs retrieved in the happy postures condition are a result of this mismatch between the whole-body posture and the hands. The presence of fists in several pictures of neutral body movements could also be accounted for by the RTs in this condition, which is comparable to happy expressions.

In conclusion, considering our results and the ones retrievable in the literature concerning emotional processing of visual stimuli, it appears that there is a gradient of complexity where facial expressions are the easiest to process, followed by emotional scenes and lastly postures, which are the hardest. Further studies will be needed in order to deeply explore this issue. The absence of significant RT differences for the IAPS stimuli is in keeping with normative data showing RT pattern for the subsets of the IAPS pictures we used in our study (“accidents” and “human attack” vs. “families and babies” and “adventure”) (Calvo and Avero, 2009). However, the analysis on accuracy during the categorization task showed an error rate higher for fear IAPS stimuli compared with happy and neutral. Although the percentage of correct recognition of the emotional content of the selected fearful stimuli is acceptable, and considering the selection of the IAPS stimuli from a specific fear-related pool (Barke et al., 2012), it appears that the possibility to select a pure set of fearful stimuli in the IAPS database might represent a limitation of this study. It should be noted that the positive IAPS category contained several stimuli depicting smiling individuals. Although happy faces tend to be efficiently recognized (Ekman and Friesen, 1982; Frank et al., 1993; Ekman, 1999; Calvo and Nummenmaa, 2008; Calvo and Beltrán, 2013), their presence in the set of complex scenes we selected was not sufficient to drive an advantage of positive IAPS stimuli relative to negative and neutral IAPS stimuli. Although a lack of difference between positive and neutral stimuli was observed also with body stimuli, further studies are needed to clarify whether the inclusion of further negative expressions (e.g., anger or disgust) could counteract the fear-specific effects we observed and favor the emergence of a positivity offset similar to that commonly reported in the literature on facial emotion recognition.

In conclusion, the results of this study show that fearful body postures are rapidly recognized and processed, probably thanks to the automatic activation of a series of central nervous system structures orchestrating the defensive threat reactions. Neurophysiological and behavioral correlates of fearful body posture processing may be valid tools for the study of psychiatric and neurodegenerative diseases. As an example, these tools might be helpful for a better comprehension of the freezing of gait phenomenon in patients with Parkinson’s disease, whose pathophysiology has been recently linked also to a dysfunction in the communication between the limbic system and the basal ganglia (Avanzino et al., 2018; Ehgoetz Martens et al., 2018; Lagravinese et al., 2018).

## Data Availability Statement

The raw data supporting the conclusions of this article will be made available by the authors, without undue reservation.

## Ethics Statement

The studies involving human participants were reviewed and approved by Ethical Committee of University of Genoa (CERA). The patients/participants provided their written informed consent to participate in this study.

## Author Contributions

AB, AA, and LA conceived and designed the experiments. AB and GL performed the experiments, analyzed the data, and wrote the manuscript. AB, LA, and MB interpreted the data. AB, LA, GL, MB, and AA drafted the article. and AB, LA, MB, and AA critically revised the article for important intellectual content. All authors contributed to the article and approved the submitted version.

## Conflict of Interest

The authors declare that the research was conducted in the absence of any commercial or financial relationships that could be construed as a potential conflict of interest.
